# The spatial epidemiology of sickle-cell anaemia in India

**DOI:** 10.1038/s41598-018-36077-w

**Published:** 2018-12-06

**Authors:** Carinna Hockham, Samir Bhatt, Roshan Colah, Malay B. Mukherjee, Bridget S. Penman, Sunetra Gupta, Frédéric B. Piel

**Affiliations:** 1 Evolutionary Ecology of Infectious Disease Group, Peter Medawar Building for Pathogen Research, Department of Zoology, University of Oxford, Oxford, UK; 2 The George Institute for Global Health, Sydney, Australia; 3 Department of Infectious Disease Epidemiology, School of Public Health, Imperial College London, London, UK; 4 Department of Haematogenetics, National Institute of Immunohaematology, Mumbai, India; 5 School of Life Sciences, Warwick University, Coventry, UK; 6 MRC-PHE Centre for Environment & Health, Department of Epidemiology & Biostatistics, School of Public Health, Imperial College London, London, UK

## Abstract

Sickle-cell anaemia (SCA) is a neglected chronic disorder of increasing global health importance, with India estimated to have the second highest burden of the disease. In the country, SCA is particularly prevalent in scheduled populations, which comprise the most socioeconomically disadvantaged communities. We compiled a geodatabase of a substantial number of SCA surveys carried out in India over the last decade. Using generalised additive models and bootstrapping methods, we generated the first India-specific model-based map of sickle-cell allele frequency which accounts for the district-level distribution of scheduled and non-scheduled populations. Where possible, we derived state- and district-level estimates of the number of SCA newborns in 2020 in the two groups. Through the inclusion of an additional 158 data points and 1.3 million individuals, we considerably increased the amount of data in our mapping evidence-base compared to previous studies. Highest predicted frequencies of up to 10% spanned central India, whilst a hotspot of ~12% was observed in Jammu and Kashmir. Evidence was heavily biased towards scheduled populations and remained limited for non-scheduled populations, which can lead to considerable uncertainties in newborn estimates at national and state level. This has important implications for health policy and planning. By taking population composition into account, we have generated maps and estimates that better reflect the complex epidemiology of SCA in India and in turn provide more reliable estimates of its burden in the vast country. This work was supported by European Union’s Seventh Framework Programme (FP7//2007–2013)/European Research Council [268904 – DIVERSITY]; and the Newton-Bhabha Fund [227756052 to CH]

## Introduction

Sickle-cell anaemia (SCA), which results from the inheritance of two copies of the sickle β-globin gene variant (β^S^), is the most common form of sickle-cell disease (SCD). SCD refers to a group of inherited disorders affecting haemoglobin^1^. Caused by a single nucleotide substitution at position 6 of the β-globin gene, its pathophysiology stems from the polymerisation of the resulting sickle haemoglobin variant (HbS), triggering a cascade of erythrocyte alterations^2,3^. Individuals with SCA experience considerable morbidity from both acute and chronic sequelae. Without effective treatment, the most severe cases can be fatal within the first few years of life^1^.

Due to improved survival and population movements, the global burden of SCA is increasing^4^, with the annual number of SCA newborns expected to increase from ~300,000 to more than 400,000 between 2010 and 2050^5^. The majority of these births occur in Sub-Saharan Africa. However, some of the highest β^S^ allele frequencies have been reported in Indian populations^6–8^, and India has been ranked the second worst affected country in terms of predicted SCA births, with 42 016 (interquartile range, IQR: 35 347–50 919) babies estimated to be born with SCA in 2010^9^.

In India, β^S^ is predominantly found amongst scheduled tribe (ST) and scheduled caste (SC) populations. These constitute the most socioeconomically disadvantaged population subgroups in the country^10^ and, according to the latest census conducted in 2011 (www.censusindia.gov.in), account for about a quarter of the Indian population. A high β^S^ allele frequency within scheduled groups is likely due to a combination of factors, including, but not limited to: (i) a potentially greater selection pressure on these groups from malaria^11^, (ii) the high rate of endogamy that is observed in them^12^, and (iii) the competitive evolutionary exclusion of β^S^ by β-thalassaemia and/or β^E^ in certain non-scheduled groups^13–15^. Heterogeneities in β^S^ allele frequency are observed within scheduled populations, with carrier frequencies ranging from ~1% to 40%^10,16^. Carrier frequencies of up to 12% have also been reported in non-scheduled groups^17,18^, although frequencies of <5% are more commonly observed^19–21^.

Although various maps of β^S^ in India and a global geostatistical map of β^S^ have previously been published^9,10,22^, a model-based national map accounting for the socio-demographic complexity of the Indian population is currently lacking. Over the last decade, public and private institutions in India have made a remarkable effort to quantify SCA prevalence in different parts of the country, ranging from village-level prevalence surveys to state-wide screening programmes (e.g. Patel *et al*., and Patra *et al*.^23,24^).

Improved knowledge of the geographical distribution and burden of SCA is essential for informing public health policies. In particular, estimates that distinguish between affected births in scheduled and non-scheduled groups may enable better assessment of the requirement for healthcare infrastructures, screening programmes and treatments, including penicillin prophylaxis, hydroxyurea and other emerging treatments^25^, for the prevention and management of SCA. Here we incorporate: (i) large amounts of recent survey data, (ii) a reproducible off-the-shelf model-based methodology, and (iii) population composition data (proportion of scheduled and non-scheduled groups) at district level, to present the first national evidence-based map of β^S^ allele frequency in India, along with sub-national estimates of the number of affected newborns expected in 2020. Coupled with ongoing extensive efforts to characterise disease survival and clinical severity in different parts of the country, this work will provide an important public health resource for developing appropriate models of care at national and sub-national levels.

## Methods

### Assembling an updated geodatabase of surveys

**Figure 1:**
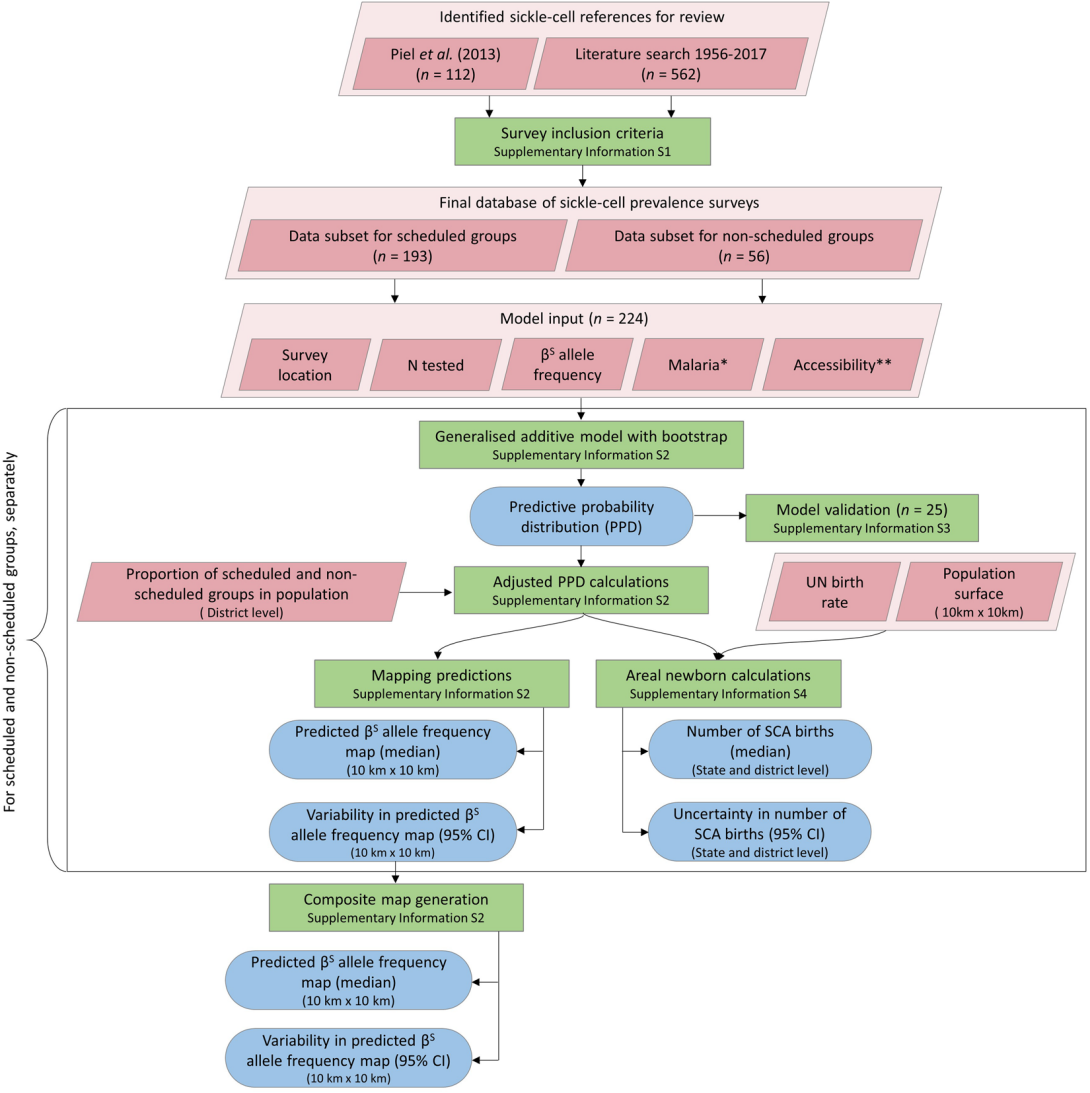
Schematic overview of database generation procedures and geostatistical modelling processes. Pink diamonds represent input data; green boxes denote methodological steps; blue rods depict model outputs. *Historical map of malaria endemicity and contemporary map of malaria. **Two urban accessibility metrics, nighttime lights and travel time to the nearest city (Supplementary Information S2).

We conducted a literature search of sickle-cell surveys in India between 2010 and 2017 as well as surveys available only in the local literature, including those older than 2010 (Fig. 1). Using procedures outlined in the Supplementary Information S1, identified references were reviewed for their suitability to contribute to the evidence-base underpinning our mapping analysis. Surveys for which there was stated or suspected selection bias in health status or SCD risk were excluded. In addition, we only included surveys that reported sample size and, at a minimum, the number of sickle-cell heterozygotes identified. Finally, only surveys that could be georeferenced to at least the district level were included. Our data were then supplemented by an earlier database published by Piel *et al*. (2013), which included studies from 1950 to 2009. The final dataset is described in detail in the Supplementary Information S1 and is available on request.

### Accounting for population composition

The ethnic composition of the Indian population is complex, with the coexistence of more than two thousand ethnic groups^26^. Under the Indian reservation system, each ethnic group is classified into one of four official social designations^27^. These, in order of decreasing socioeconomic deprivation, are: (i) Scheduled Tribes, (ii) Scheduled Castes, (iii) Other Backward Classes (OBCs), and (iv) General Classes (GCs). While specific ethnic information is not consistently reported in published surveys, it is usually relatively straightforward to identify whether the studied populations were scheduled or non-scheduled. To account for population composition, surveys were included irrespective of the ethnicity of the study sample and categorised as “scheduled” or “non-scheduled” (Supplementary Information S2). Surveys for which the ethnicity and/or social status of the sample was unknown or mixed were excluded.

### Analysis of associations between covariates and β^S^ allele frequency

Univariate linear regression was used to examine the association between social group and β^S^ allele frequency to confirm that there was indeed a difference in β^S^ allele frequency between scheduled and non-scheduled surveys. The dataset was then divided into two data subsets: scheduled and non-scheduled.

We used a generalised additive modelling (GAM) approach to examine associations between the observed allelic counts from survey data with a series of predictor variables (or covariates): (i) geographical location, given by latitude and longitude in decimal degrees, (ii) historical rates of malaria, taken from two separate sources^28,29^, (iii) contemporary rates of malaria^30^, and (iv) two urbanisation proxies (Supplementary Information S2).

A description of the model procedures is provided in the Supplementary Information S2. We divided each dataset (scheduled and non-scheduled) into a training dataset, comprising 90% of the data points, and a semi-random 10% hold-out dataset (Supplementary Information S3). For each training dataset, we used a backward stepwise selection procedure, starting with a full model that included all covariates, to decide upon a final GAM. A two-dimensional smoother was used for the geographical effect to account for spatial autocorrelation^31^. The Generalised Cross Validation (GCV) score, mean squared error (MSE) and Akaike Information Criterion (AIC) score were used as selection criteria, along with *p*-values for individual covariates.

The predictive ability of each model was assessed by comparing model predictions with the observed β^S^ allele frequencies for the corresponding hold-out dataset (Supplementary Information S3). The mean error (ME) and mean absolute error (MAE) were calculated as an indication of the model’s overall bias and accuracy, respectively.

### Creating a map of β^S^ allele frequency

For each dataset, the final fitted model was used to predict β^S^ allele frequency at unsampled locations and generate a map at 10 km × 10 km resolution (Supplementary Information S2). We then adjusted our predictions using census data on the proportion of scheduled and non-scheduled populations at the district level (*n* = 666) (www.censusindia.gov.in) by multiplying them together. These adjusted maps were combined to generate a composite map of β^S^ allele frequency in India that incorporated information from scheduled and non-scheduled populations.

Bootstrap resampling (with replacement) of the two datasets was performed for 2500 iterations to generate a predictive probability distribution for each pixel, from which the median could be calculated along with the 95% confidence interval (95% CI). The 95% CI was used as a measure of the variation in the models’ predictions at each 10 km × 10 km location. We would like to make clear that, particularly in areas where data are absent (e.g. in Haryana, Uttarakhand, many of the northeastern states and parts of southern India), the 95% CI should not be interpreted as the level of uncertainty in our estimation of the *true* frequency of β^S^ in the population; rather it is a reflection of the models’ consistency in predictions as a result of no data.

### Estimating number of newborns affected

The number of newborns with SCA in India in 2020 was estimated for scheduled and non-scheduled populations separately by pairing our 10 km × 10 km maps of β^S^ allele frequency with high-resolution birth count data (described in the Supplementary Information S4). The predicted number of newborns with SCA (N_SCA_) was based on Hardy-Weinberg assumptions, so that N_SCA_ is given by *Bq*^2^, where *B* is the number of births in each pixel and *q* is β^S^ allele frequency^32^. To calculate areal estimates, estimates in each pixel were generated for each bootstrap repetition of the model and summed across all pixels falling within an administrative unit. This generated a predictive probability distribution for the number of affected newborns in each unit, which was used to calculate the median and 95% CI for the newborn estimates. Uncertainty measures incorporated uncertainty in both the behaviour of the GAM predicting β^S^ allele frequency and the birth count data (Supplementary Information S4). Again, these uncertainty measures should be interpreted with the caveat that the GAM makes consistently low predictions of β^S^ allele frequency in the absence of data. Therefore, narrow 95% CIs in areas where data is absent should not be interpreted as certainty in the absence of sickle-cell in those regions, but rather consistency in what this method predicts for them.

All statistical analyses were performed in R using the ‘mgcv’ package (with version R 3.3.2).

## Results

### Prevalence survey database

**Figure 2:**
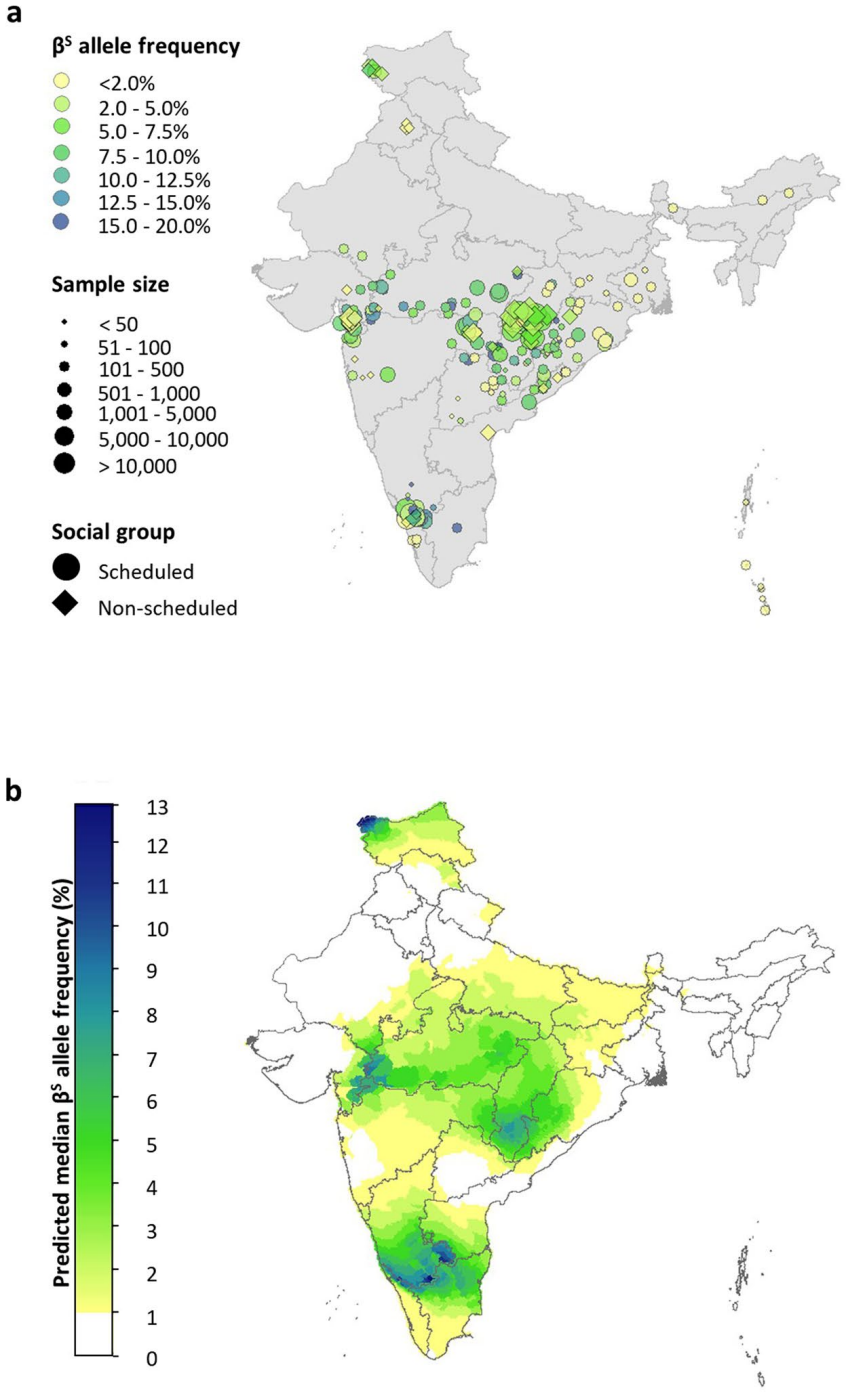
(**a**) A map of the sickle-cell surveys included in our database (n = 249). Data points are coloured according to the β^S^ allele frequency reported in the study sample. The size of the data points relates to their sample size. A spatial jitter of up to 0.3° latitude and longitude decimal degrees coordinates was applied to improve visualisation of the data. (**b**) Map of median predicted β^S^ allele frequency estimates at a resolution of 10 km × 10 km. State boundaries are displayed in dark grey.

Our final evidence-base consisted of 249 surveys from 75 sources, spanning 141 spatially unique sites (Fig. 2a). Surveys were conducted in 18 of the 36 Indian states and union territories. More than half (60.64%) fell within four states: Gujarat (*n* = 29), Maharashtra (*n* = 32), Odisha (*n* = 37) and Chhattisgarh (*n* = 53) (Fig. S2a). Scheduled populations were the most extensively studied, with 171 surveys carried out amongst STs, 18 amongst SCs and four amongst the two groups combined. Thirty-one surveys targeted populations belonging to OBCs, 24 were carried out in GCs and one in OBCs and GCs together. The number of individuals sampled was 1 300 719 (compared to 34 382 in Piel *et al*. - an almost forty-fold increase) and sample size ranged from 2 to 150 988. Some of the very small samples (e.g. *n* = 2) come from surveys that were carried out across multiple ethnic groups but the β^S^ allele frequency reported separately for each. Mean sample size was 5224 and the median 244.

### Analysis of associations between covariates and β^S^ allele frequency

A univariate analysis of the relationship between β^S^ allele frequency and social group (scheduled or non-scheduled) for the whole dataset revealed a *p*-value < 0.0001. The dataset was separated into scheduled and non-scheduled datasets (*n* = 193 and *n* = 56, respectively), and divided into training and hold-out datasets. For both, only geographic location was included in the final model (Table 1). The *p*-value for the two-dimensional smooth term was < 0.0001 for both. The large effective degrees of freedom (edf) indicate a highly non-linear relationship between geographic coordinates and β^S^ frequency. The R^2^ value was greater for the non-scheduled dataset (R^2^ = 0.68) than for the scheduled dataset (R^2^ = 0.52), suggesting that a simple spatial model explains a larger proportion of the variance in the former.

**Table 1:** Summary results of the selected GAM for the scheduled and non-scheduled training datasets.

	Estimate	*p*-value
**Scheduled dataset (** ***n*** ** = 174)**		
**Intercept**	−3.0898	< 0.0001
**Smooth term**	**edf**	***p*** **-value**
***f*(lat**, **long)**	22.1800	<0.0001
R^2^ = 0.5170		
GCV = 1.0038		
MSE = 0.7555		
AIC = 495.9284		
**Non-scheduled dataset (** ***n*** ** = 50)**		
**Intercept**	−4.0709	<0.0001
**Smooth term**	**edf**	***p*** **-value**
***f*(lat**, **long)**	10.0900	<0.0001
R^2^ = 0.6890		
GCV = 1.0728		
MSE = 0.6639		
AIC = 150.4534		

The intercept, smoothing term f and its corresponding p-value, adjusted R^2^, Generalised Cross Validation (GCV) score, mean squared error (MSE) and Akaike Information Criterion (AIC) are given.

### β^S^ allele frequency map and prediction uncertainty

Predicted maps for the sub-populations were generated separately (Supplementary Figs S5 and S6) and then paired, together with district-level data on the proportion of scheduled and non-scheduled groups, to create a final composite map of β^S^ allele frequency (Fig. 2b). A map showing the 95% CI associated with each pixel when bootstrapping is used to explore how consistently the GAM performs is provided in Supplementary Fig. S7.

The highest predicted allele frequencies (up to 10%) where consistency in model predictions was high (95% CI ≤ 5%) were found in a belt stretching across central India, extending from southeastern Gujarat to southwestern Odisha. Within this belt, lower allele frequencies of 2–6% were predicted in the Nagpur Division of Maharashtra and still lower frequencies of 1–2% in the Konkan and Pune Divisions. Similarly heterogeneous frequencies were predicted in southeastern Rajasthan, Gujarat and Odisha. Large parts of Madhya Pradesh and Chhattisgarh were predicted to have a β^S^ allele frequency ≥4%. For all the aforementioned regions, the model performed largely consistently when the data were bootstrapped (95% CI ≤ 5%), with some exceptions (Fig. S7). Allele frequencies of ~12% were predicted in northwestern Jammu and Kashmir, although there were large inconsistencies in the model predictions for this region (95% CI ~10%). Very low frequencies (<1%) were predicted in the whole northeastern region, although with even greater variability in the model’s behaviour in some areas (e.g. 95% CIs of ≥80% in parts of Assam). Predictions in southern India were also associated with high variability in model behaviour (95% CIs >20%). Finally, in areas where there were no data available, the model consistently predicted low frequencies of <1%. Examples include Haryana, Uttarakhand, Uttar Pradesh, Bihar, the central part of Karnataka and Andhra Pradesh and some of the northeastern states.

### Validation statistics

We compared predictions generated using the training data with known values in the hold-out subset. Our comparison revealed a mean error in allele frequency prediction of −4.3% and −0.8% in the scheduled and non-scheduled groups, respectively. The relatively high mean error for scheduled populations can in part be explained by the large amount of heterogeneity in the observed data. The predicted allele frequencies for non-scheduled populations were only slightly overestimated. The mean absolute error was 4.6% and 0.8%, respectively. The predicted allele frequencies in the scheduled and non-scheduled maps are therefore slightly overestimated (i.e. mean errors were mostly positive).

### Estimates of newborns affected in scheduled and non-scheduled populations

**Figure 3:**
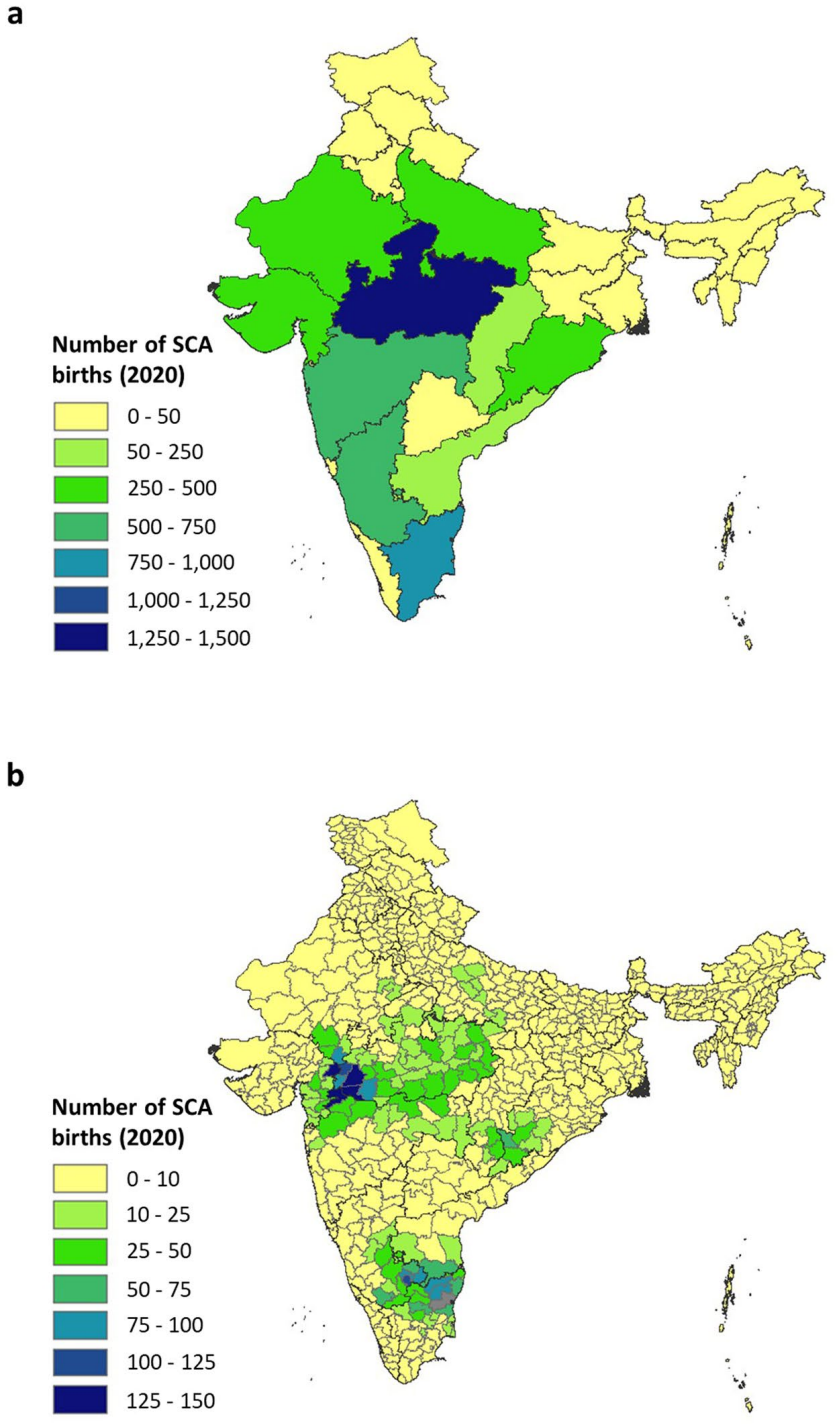
Map of the estimated number of scheduled newborns born with SCA in India, (**a**) by state and, (**b**) by district, in 2020. The medians of the predictive probability distribution of the areal estimates are displayed. The district shaded grey in Tamil Nadu in (b) is that where the 95% CI was very large (>1000). State boundaries are displayed in dark grey and district boundaries in light grey.

**Figure 4:**
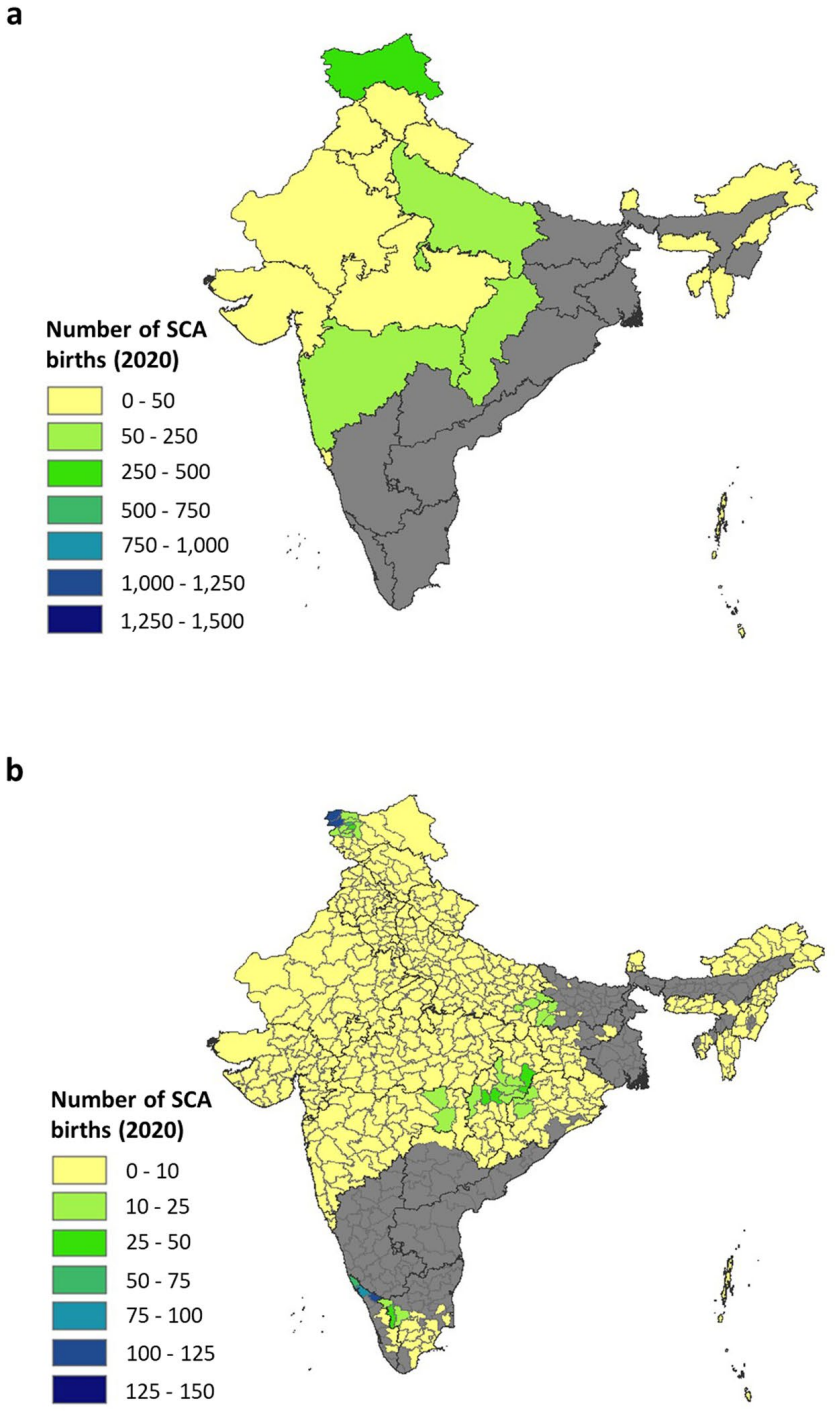
Map of the estimated number of non-scheduled newborns born with SCA in India, (**a**) by state and, (**b**) by district, in 2020. The medians of the predictive probability distribution of the areal estimates are displayed. The states and districts shaded grey are those where our estimates were highly variable (95% CI > 10 000 and > 1000, respectively (Supplementary Figure S9). State boundaries are displayed in dark grey and district boundaries in light grey.

The absolute burden of β^S^ depends on both β^S^ allele frequency and population size. We generated state- and district-level estimates of the number of newborns with SCA in 2020 for scheduled and non-scheduled groups, by pairing the respective adjusted predicted allele frequency maps with birth data (Figs 3 and 4). The 95% CI for these estimates are provided in Supplementary Figs S8 and S9.

For scheduled groups, the highest number of affected newborns was predicted in Madhya Pradesh (1475 [95% CI: 753–3307]). Maharashtra, Gujarat, Odisha and Chhattisgarh were predicted to have 503 (95% CI: 270–870), 436 (95% CI: 263–711), 257 (95% CI 149–247) and 230 (95% CI 120–493) SCA newborns, respectively. The second highest number of affected newborns was predicted in Tamil Nadu (846 [95% CI 30–9268]), although this state also had the largest 95% CI associated with that prediction (95% CI ~9000). A burden of <50 newborns was predicted in Jammu and Kashmir, Punjab, Telangana, Jharkhand and West Bengal, reflecting the low observed β^S^ allele frequencies and/or small population size in these states compared to higher-burden states. In states where data were absent (e.g. Uttar Pradesh, northern Karnataka and many of the northeastern states), estimates represent the minimum estimate for each state (Fig. 3a). This is due to the absence of data preventing higher β^S^ allele frequencies from being predicted.

Figure 3b shows the number of newborns estimated at district level. For the majority of districts (82%), 10 or fewer SCA cases were predicted. Again, for regions where no data were available, these estimates must be interpreted as minimum estimates. Districts with the highest estimated number of SCA newborns in scheduled groups were found on or close to the border of Gujarat, Maharashtra and Madhya Pradesh and include: Dahod district in Gujarat (146 [95% CI: 79–265]), Nandurbar district in Maharashtra (145 [95% CI: 73–266]) and Dhar and Barwani districts in Madhya Pradesh (144 [95% CI: 80–245] and 139 [95% CI: 70–250], respectively). A hotspot of districts with a predicted burden of 75 or more cases was also predicted at the border of Tamil Nadu, Karnataka and Andhra Pradesh although the 95% CI for these states was larger (95% CI > 500; Supplementary Fig. S8b).

For non-scheduled groups, sensible predictions could not be made for a third of Indian states, including Kerala, Karnataka, Tamil Nadu, Andhra Pradesh, Telangana, Odisha, Jharkhand, Bihar, West Bengal, Assam and Manipur (Fig. 4a). For all these states, the 95% CI exceeded 10 000 (Supplementary Fig. S9a). For states where the data allowed calculation of sensible estimates, Jammu and Kashmir was predicted to have the highest number of SCA newborns (461 [95% CI: 127–1542]), followed by Chhattisgarh (249 [95% CI: 129–542]), Uttar Pradesh (153 [95% CI: 19–5186]) and Maharashtra (91 [95% CI: 43–4051]). The remaining states were all estimated to have 50 or fewer SCA newborns, which are minimum estimates given the absence or paucity of data.

At the district level, for 172 of the 666 districts, our estimates were deemed to be too variable (95% CI ≥ 1,000) to be meaningful (Fig. 4b and Supplementary Fig. 9b). Of the remaining districts, those with the highest predicted burden were Kupwara (125 [95% CI: 27–431]) and Baramulla (123 [95% CI: 32–400]) in Jammu and Kashmir and Wayanad (124 [95% CI: 0–205]) and Kannur (92 [95% CI: 0–771]) in Kerala. Four hundred and fifty-five districts were predicted to have 10 or fewer SCA newborns due to data for non-scheduled groups being absent for many of the districts.

## Discussion

The patterns in our maps are consistent with previous survey^10,22^ and continuous maps^9^, with the lowest allele frequencies predicted in the northeastern part of the country, the highest frequencies across a central belt, an area of high allele frequency in southern India, and a heterogeneous distribution of the β^S^ allele across the whole country. However, our database and map also suggest a hotspot in northwestern Jammu and Kashmir, which stems from a survey carried out by Fareed *et al*.^17^ in Rajouri and Poonch districts in which β^S^ allele frequency ranged from 2.69% to 8.75%. This may warrant further investigation as β^S^ is typically considered to occur at low frequencies in the north of the country.

An important difference between our map and previous maps is the inclusion of social status in our analysis. Our findings highlight that our current knowledge of the distribution of β^S^ in India is based on an evidence-base that is heavily biased towards scheduled groups, with close to 80% of the data coming from scheduled populations. Whilst it is important to assess the burden amongst the socioeconomically deprived scheduled groups, which experience some of the highest β^S^ frequencies in the country, the generation of reliable estimates for the whole Indian population requires understanding the epidemiology of SCA amongst non-scheduled groups too, for three reasons: (i) current surveys reveal a not insignificant amount of heterogeneity in sickle-cell frequency in non-scheduled groups, with observed allele frequencies ranging from 0% to 12%, (ii) non-scheduled groups account for 75% of the Indian population (i.e. 991 327 500 individuals in 2020, including 23 943 203 newborns, assuming a birth rate of 18.1 per 1000 population), and (iii) together, this means that, without more data for non-scheduled groups, any estimate of the number of SCA newborns in this large subset of the population would be associated with considerable imprecision and uncertainty.

Since the publication of the global β^S^ allele frequency map in 2013, there has been a surge of prevalence surveys, some of considerable size (>1 million individuals), carried out in India. The inclusion of data from recent screening programmes and population surveys more than doubled the number of data points in our evidence-base (*n* = 249 compared with *n* = 112 in Piel *et al*.). However, we found considerable geographical overlap between the surveys identified here and those used in Piel *et al*. (2013). This added heterogeneity to the existing evidence-base, which is reflected by the lower precision of our estimates in areas where data were abundant. However, we believe that this better reflects the true heterogeneity of β^S^ allele frequency in the worst affected parts of the country. For areas where heterogeneity is very high (e.g. Tamil Nadu), it may be necessary to scale future geospatial analyses down to the within-state level.

Our analyses also demonstrate that, for a national allele frequency map such as the one presented here, an uneven spread of surveys makes it hard to generate predictions in areas where data are absent. Although the assumption that frequencies are likely to be low in unsampled areas may seem reasonable, we found evidence that this is not always true. The stringent inclusion criteria used in this study, including georeferencing at the district level and unambiguous scheduled status of the study sample, meant that many surveys were excluded from the analysis. A few of these excluded surveys offer data in areas where no surveys meeting our inclusion criteria were conducted. For instance, analyses carried out in the Tharu tribal group of the Terai region of Uttar Pradesh revealed a β^S^ allele frequency of 10%^33^. This survey could not be georeferenced to the district level and was therefore excluded from the present study. Without any data indicating the presence of β^S^ in the Terai region, geospatial analysis cannot predict it. Developing methods that can combine data of different quality (e.g. presence/absence of β^S^ versus β^S^ frequency; georeferencing to different administrative levels; known versus unknown scheduled status of the study sample) within a single unified analysis remains an ongoing challenge.

There are some limitations to our map and newborn estimates. First, the precision of a model-based map and estimates are determined by the available data, which is non-randomly distributed. Second, the categorisation of ethnic groups into two categories is reductionist; even within the groups, there is extensive heterogeneity in β^S^ allele frequencies^22,34^. The inclusion of more specific ethnicities in the analysis would have resulted in a more tailored map and estimates; however, detailed data on the distribution of all ethnic groups in India are limited. In addition, our estimates do not account for consanguinity, due to there being no fine-scale data on consanguinity for the country. It is therefore likely that our estimates are an underestimate of the true burden.

Our analyses highlight some of the complexities and heterogeneities of the populations living in the Indian subcontinent. They strongly point towards the need for careful planning by the upcoming National Programme for Control and Care of Haemoglobinopathies to generate data that will lend itself to improved precision in current estimates. For example, within the high-frequency states, ongoing surveys would be beneficial to assess the impact of screening and other interventions. Data in non-scheduled populations is also needed, particularly in southern India, where uncertainty in our newborn estimates is high. The reporting of the scheduled status of sample individuals in future surveys will also be important in order to avoid a trade-off between the density of data points and the inclusion of social status in future mapping analyses, as has occurred in this study.

Our analyses also reflect some of the challenges in defining optimum public health strategies in such a setting. Focusing on high-frequency hotspots of scheduled populations might be the most cost-effective option but would neglect a large number of SCA individuals in low-frequency areas of primarily non-scheduled populations. Well-designed epidemiological surveys will be crucial to further assess the prevalence and burden of SCD in India and the impact of chosen public health interventions. Recent low-cost point of care testing devices will greatly facilitate this.

## Electronic supplementary material

Supplementary Information

Source data for map of the district-level distribution of scheduled groups in India

## Data Availability

The datasets generated and analysed during the current study are available from the corresponding author on reasonable request.
