# Supplementary material for: The spatial epidemiology of sickle-cell anaemia in India
Source: Sci Rep. 2018 Dec 6;8:17685. doi: 10.1038/s41598-018-36077-w (PMC6283872; doi:10.1038/s41598-018-36077-w)
Supplement: Supplementary file 1 — Supplementary Information [file 41598_2018_36077_MOESM1_ESM.pdf]

The spatial epidemiology of sickle-cell anaemia in India

Supplementary Information

Carinna Hockham (DPhil)[1,2]*, Samir Bhatt (DPhil)[3], Roshan Colah (PhD)[4], Malay B Mukherjee (PhD)[4], Bridget S Penman (DPhil)[1,5], Sunetra Gupta (PhD)[1] & Frédéric B Piel (PhD)[1,6]

1. *Evolutionary Ecology of Infectious Disease Group, Peter Medawar Building for Pathogen Research, Department of Zoology, University of Oxford, Oxford, UK*
2. *The George Institute for Global Health, Sydney, Australia*
3. *Department of Infectious Disease Epidemiology, School of Public Health, Imperial College London, London, UK*
4. *Department of Haematogenetics, National Institute of Immunohaematology, Mumbai, India*
5. *School of Life Sciences, Warwick University, Coventry, UK*
6. *MRC-PHE Centre for Environment & Health, Department of Epidemiology & Biostatistics, School of Public Health, Imperial College London, London, UK*

*Corresponding author: Carinna Hockham (chockham@georgeinstitute.org.au)

Supplementary Information 1: Assembling a geodatabase of sickle-cell prevalence surveys

Library assembly

Online searches of two major bibliographic databases, PubMed (http://www.pubmed.gov) and Scopus (http://www.scopus.com) were performed to identify published surveys of β^S allele frequency carried out in India since 2010. To ensure consistency with the search strategy used in Piel *et al.* (2013),[1] the same keyword string was used: “sickle cell” or “haemoglobin S” or “hemoglobin S” or “Hb S”. We also included the search term “India” to make it country-specific and limited our search to between 2010 and 2016. Our final search on 14 October 2016 yielded 502 references across all three databases, which were imported to a bibliographic management software (Endnote X7.7.1, Thomson Reuters, Carlsbad, CA, USA). Twenty-four duplicates were identified and manually removed, leaving a total of 478 references to be reviewed. Unpublished sources of data and references available only in the local literature (n = 84) were identified through our UK-India collaboration, supported by the Newton Bhabha Fund, and reviewed in the same manner. Finally, the dataset used by Piel *et al.* (2013)[1] in their global sickle-cell mapping study was clipped to India and merged. All additional sources from which data were abstracted are listed in the Supplementary Bibliography S1. Sources included in the earlier database are listed in Piel *et al.* (2013).

Data inclusion criteria and abstraction process

The identified sources were reviewed in detail by one of the authors (CH) using a specific set of inclusion criteria, which were outlined in a pre-defined protocol. This ensured objectivity and consistency in the data abstraction process. A random sample of 10% of the references were reviewed and checked by a second author (FP), and the survey details extracted by the two authors compared.

Survey representativeness

Only surveys that were conducted among representative population samples were included in the database. Two key considerations when determining the representativeness of a sample were: (i) the source population from which the sample was taken, e.g. pregnant women, newborns, community, etc., and (ii) the sampling methodology used, e.g. random, consecutive, unselected or universal. All family-based studies as well as those conducted amongst patients (e.g. malaria patients or general outpatients) or suspected cases of haemoglobinopathies were deemed to be inherently biased and thus immediately excluded. However, surveys that took place amongst newborns, pregnant women, children and adolescents or in the community, although unbiased in terms of their source population, were scrutinised for their sampling methodology. Those with stated or suspected bias in their selection method were excluded. For instance, studies that took place in the community but which required voluntary participation without any prior education or awareness campaign were excluded due to a potential bias, for example towards individuals with an affected family member.

Ethnicity and social grouping

Previous efforts to map β^S have excluded surveys that target specific ethnic groups due to their lack of representativeness of the general population. However, given the unique population structure of India in the form of highly endogamous population groups[2] and the well-known heterogeneities in β^S frequency across ethnic and social groups,[3,4] we included surveys irrespective of the ethnicity of the study sample, and accounted for this in the mapping analysis (Supplementary Information S2). In India, ethnicity is inherently linked to social caste.[3] As such, we categorised ethnic groups according to their social category as defined by the Government of India. The four designations used were: (i) Scheduled Tribe (ST), (ii) Scheduled Caste (SC), (iii) Other Backward Class (OBC), and (iv) General Class (GC). State-wise lists of

communities belonging to the STs, SCs, and OBCs were sourced from the websites of the Ministry of Tribal Affairs (http://tribal.nic.in), the Ministry of Social Justice and Empowerment (http://socialjustice.nic.in) and National Commission for Backward Classes (http://www.ncbc.nic.in), respectively. Any ethnic groups that were not included in these lists were assumed to belong to the GC category. Subsequently, the data were split into two subsets: (i) surveys carried out in scheduled populations (i.e. STs and/or SCs), and (ii) surveys carried out in non-scheduled population (i.e. OBCs and/or GCs). Those surveys that did not specify the ethnic origin or social grouping of the study sample or included a mixture of scheduled and non-scheduled individuals were entered into the database but excluded from our mapping analysis.

Survey diagnostic methods

There are several methods available for the diagnosis of SCT and/or SCD.[5-7] These include the sickling test, solubility test, haemoglobin electrophoresis and high performance liquid chromatography (HPLC). The sickling test is based on the principle that affected red blood cells become sickle-shaped when exposed to a low oxygen tension. A blood sample is mixed with sodium metabisulphite (an oxygen scavenger) and examined under a microscope. By contrast, in a solubility test, the red blood cells are lysed to release the haemoglobin into the blood plasma. Normal haemoglobin is soluble in the blood plasma, whilst sickle haemoglobin is not and forms small crystals that cause the blood plasma to become turbid.[8] In India, the most commonly used methods are sickling test and HPLC, depending on the resources available.[6] Given the good reliability of most diagnostic methods for β^S,[5] no strict inclusion criteria with regards to diagnostics were applied. However, key information regarding the precise diagnostic algorithm used in a survey was recorded. This included the presence or absence of an initial screening stage and the type of confirmatory diagnostic test used. Where multiple confirmatory

diagnostic tests were used, the most reliable method – usually HPLC or, in some cases, DNA sequencing – was recorded. This information is available on request.

Sample size and β^S allele frequency

An important prerequisite to the inclusion of a survey in the database was the clear reporting of both the sample size and the number of AS individuals identified. When provided, the number of SCA individuals and any compound heterozygotes (e.g. co-inheritance of β^S with β-thalassaemia or with another structural variant such as β^E) were also recorded. Occasionally, surveys directly reported the frequency of the β^S allele without a breakdown of the different genotypes. These were also included, but only if sample size was provided.

Genotype counts were used to calculate the allele frequency of β^S, a key input for our model, using the formula:

$$q = \frac{(2 * n_{SCA}) + n_{SCT}}{2N}$$

where q is the β^S allele frequency, n_{SCA} and n_{SCT} are the number of β^S homozygotes and heterozygotes or compound heterozygotes, respectively, and N is the size of the study sample. A simplifying assumption of this calculation is that the populations are at Hardy-Weinberg equilibrium (HWE).[9,10]

Sample size varied enormously between surveys. We did not place any constraints on sample size, but rather accounted for it in the statistical model by weighting the contribution of each survey to the fitted model according to its size. As a result, the contributions of very small samples were down-weighted whilst those of larger samples were strengthened.

Georeferencing

We applied an inclusion criterion of spatial specificity, whereby only those surveys that could be georeferenced to at least the second administrative level were included. In India, this corresponds to the district level (https://censusindia.gov.in). Various geopositioning gazetteers were used to identify the latitude and longitude of all included surveys as precisely as possible. Where a survey described a specific sampling site, the geographic coordinates of that site were used. For surveys only reporting the district in which they were conducted, the centroid of the district was identified and recorded. In instances where the presented survey data came from multiple sites, the centroid between all the survey sites was determined using the Geographic Information Systems (GIS) software, ArcGIS Desktop (ArcMap 10.4.1, ESRI Inc., Redlands, CA, USA). No information on the spatial extent of the surveys was recorded, as this information was rarely available.

An advantage of our modelling approach, which is described in detail in Supplementary Information S2, is that all spatial duplicates could be retained, thereby maximising the size of the evidence-base for the mapping model.

The final sickle-cell survey dataset

Of the 562 sources reviewed as part of the updated literature search, 75 met the inclusion criteria outlined above and contributed 158 data points to the evidence-base. As a result, the inclusion of surveys carried out in India since 2010, as well as surveys available only in the local literature, more than doubled the size of the previous evidence-base for India upon which a continuous map of β^S frequency was constructed (n = 91 in Piel *et al.* compared to n = 249 in this study). Apart from surveys in the north of India (Punjab and Jammu and Kashmir), the additional data increased the number of data points in previously surveyed areas

(Supplementary Figure S1a), rather than expanding the spatial distribution of the data points included in the database. The temporal distribution of the surveys for each state is shown in Supplementary Figure S1b, along with information on the database in which they were initially recorded and their sample size. Very few surveys were missed by Piel *et al.*'s previous database, illustrating the comprehensiveness of the systematic search and source review process used by them and in this study. The additional surveys conducted prior to 2010 that were found came from 12 sources that could only be accessed from local journals and centre reports.

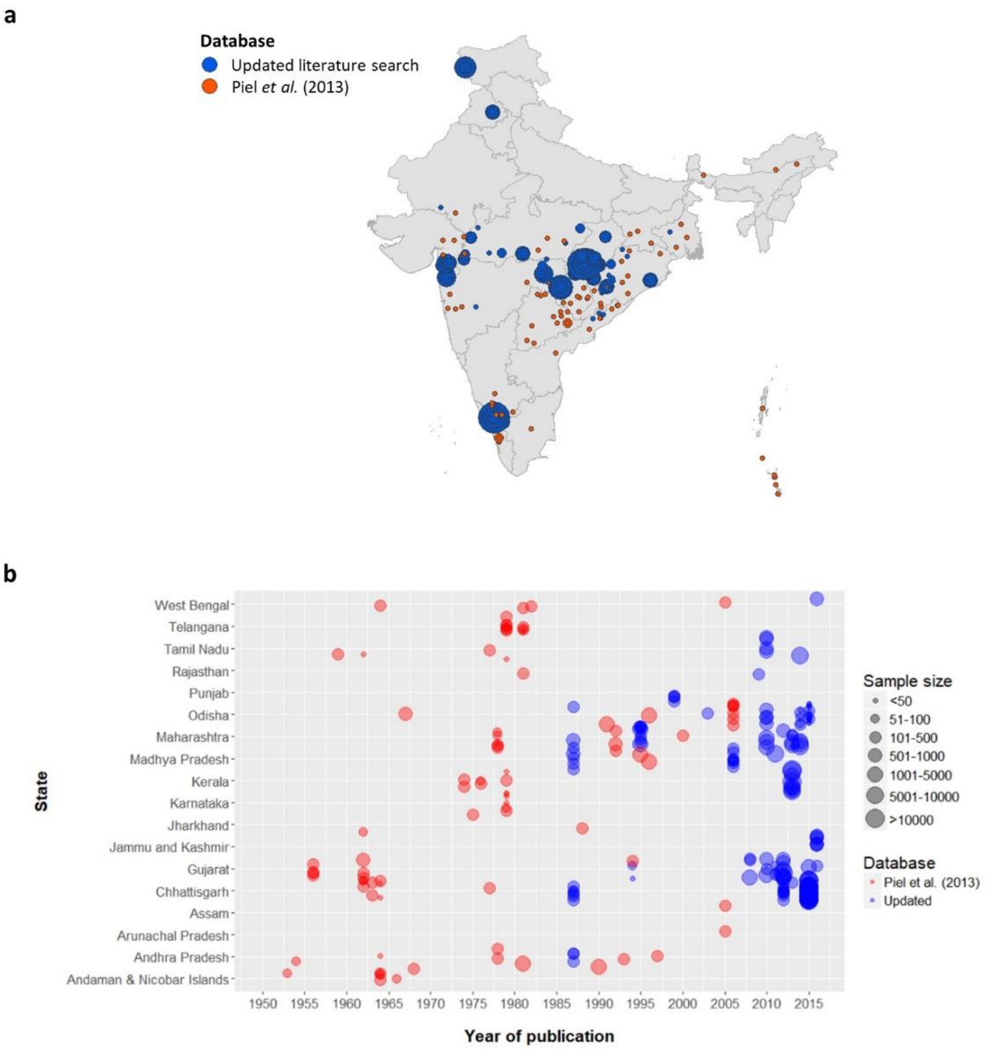

Supplementary Fig. S1 Spatial and temporal distribution of sickle-cell surveys in the database. (a) Map showing the spatial distribution of the data points, coloured according to the searches in which they were included. Red circles represent surveys that were originally identified in the geodatabase by Piel and colleagues;[1] blue circles depict surveys obtained through our updated literature search. Concentric circles are used to indicate multiple surveys from the same geographical location, with each circle representing a single survey. Here, the positioning of the data points on the maps reflects their true geographical coordinates. (b) Temporal distribution and sample size of the β^S surveys by state. Again, the colour of the data points indicates the database in which they were originally included, while size depicts the sample size of the survey

The spatial distribution of surveys and the survey count by state are shown in Supplementary Figure S2. Surveying effort was unevenly distributed; over half of the surveys fell within just four states (Gujarat, Maharashtra Odisha and Chhattisgarh) and some of the largest surveys were carried out in two of these states (Maharashtra and Chhattisgarh). Nevertheless, this reflects the areas of highest concern with regards to SCA.[4,6] The total number of individuals sampled was 1 300 719 with 88.1% of these sampled in these four states.

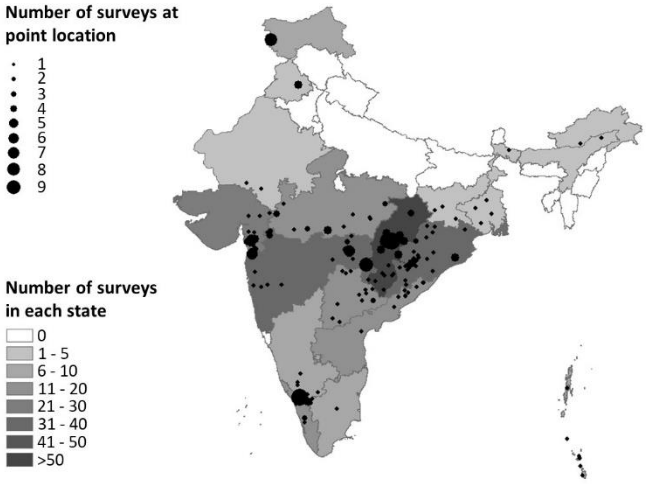

Supplementary Fig. S2 Survey count by state. The colour intensity of the state indicates the number of surveys that were carried out in it, whilst circle size indicates the number of surveys that were carried out at each point location within the state

Surveys were carried out in a range of specific population groups, including newborns (*n* = 25), schoolchildren and adolescents (*n* = 63) and pregnant women and/or their husbands (*n* = 1). However, the majority of the surveys were carried out in community samples (*n* = 133). Twenty-seven surveys lacked information on their target population group (pregnant women, newborns, etc.), but did not indicate any potential bias and so were retained. Scheduled tribes were the most studied group, following by scheduled castes and other backward classes.

Some heterogeneity in the diagnostic algorithm used was also observed. However, the vast majority of the surveys used HPLC as the confirmatory diagnostic test, with less than half of these incorporating an additional screening stage.

Supplementary Information 2: Creating a β^S allele frequency map

Overview of generalised additive models (GAMs)

To generate a continuous map of β^S allele frequency in India, we employed a generalised additive modelling approach. Generalised additive models (GAMs) comprise a collection of nonparametric regression techniques performing regression inference on complex, non-linear, relationships between a given response variable and multiple predictor variables.[11] The advantage of using GAMs over more common linear models is that they can create extremely flexible functional relationships while effectively regularising the objective function - that is, they automatically trade off over/under fitting to provide the best estimation of the underlying predictive patterns.[12] In addition, unlike more black box methods, such as gradient boosting machines,[13] the models are easily interpretable due to the contribution of each independent variable to the prediction being explicitly encoded.

For a given response $Y \in R$ and D associated predictors $X \in R^D$, the GAM estimates an unknown function $Y = f(x)$ through an additive series of smooth functions, i.e.

$$g\big(E(Y)\big) = a + s_1(x_1) + s_2(x_2) + \cdots + s_n(x_n) + \cdots + x_p$$

where E(Y) denotes the expected value of the response and g(Y) denotes the link function that allows for the modelling of non-Gaussian likelihoods.[11] The terms $s_1(x_1),\ldots,s_n(x_n)$ denote smooth, nonparametric thin-plate spline functions. GAMs can also be generalised to multiple dimensions to model the interactions between two predictor variables such as spatial coordinates. The general principles of generalised additive modelling have been described previously by Hastie and Tibshirani (1987)[14] and West (2012).[15]

Model covariates: malaria

Whilst the malaria hypothesis is widely accepted and supported by high-quality and varied evidence,[16] the geographical relationship between sickle-cell and historical malaria remains unresolved in the Indian context. This is presumably due to a myriad of other factors influencing the distribution of β^S here, including population structure, consanguinity and migration. To explore this relationship using our GAM approach, we sourced two maps of the pre-control distribution of malaria in India (Supplementary Figure S3). The first was a global map produced by a team of researchers in the 1960s and represents the distribution of malaria when it was at its highest, circa 1900 (Supplementary Figure S3a).[17] The authors synthesised information and data on multiple malariometric indices from a range of sources and combined this with expert opinion as well as temperature and rainfall data to generate the map. To suit the purposes of our study, the map, which was previously used by Piel *et al.* (2010), was clipped to India in ArcGIS Desktop. The second map was produced by Sir Patrick Hehir in 1927 and is specific to India (Supplementary Figure S3b).[18] The map was previously digitised by Dr Katherine Battle from the Malaria Atlas Project, who shared it with us for this study. Whilst there is little information on the evidence-base upon which the map was generated, to our knowledge it is the only available pre-control map of malaria specifically for India. Coupled with the additional detail provided by the map compared to that from Lysenko et al. (1968), this justified its inclusion in our analysis.

To gain insight into the relationship between sickle-cell in India and present-day malaria, we also obtained a contemporary map of malaria from the Malaria Atlas Project map repository (MAP, www.map.ox.ac.uk) (Supplementary Figure S3c). Generated using a Bayesian geostatistical model that incorporates data from parasite rate surveys as well as from environmental covariates, the map shows the mean value of the range of age-standardised

Plasmodium falciparum parasite rates predicted for each location, at a resolution of 1km x 1km.[19]

Collinearity refers to the non-independence of predictor variables such that one predictor could be reasonably well modelled as a function of another. This can cause problems for parameter estimation, leading to the incorrect identification of relevant predictors in the model. To test for an association between the two historical malaria maps, both of which presented ordered categorical malaria epidemiological data, we used univariate linear regression, which revealed a *p*-value of <0.0001. Univariate linear regression models were also used to examine the collinearity between contemporary parasite rates and historical endemicity classes. A very small *p*-value of <0.0001 was observed in the test for an association between contemporary malaria and the map by Hehir, whilst a *p*-value of 0.24 was found in the test for an association between the maps by Lysenko *et al.* and MAP. The latter two maps were therefore retained for the model selection process.

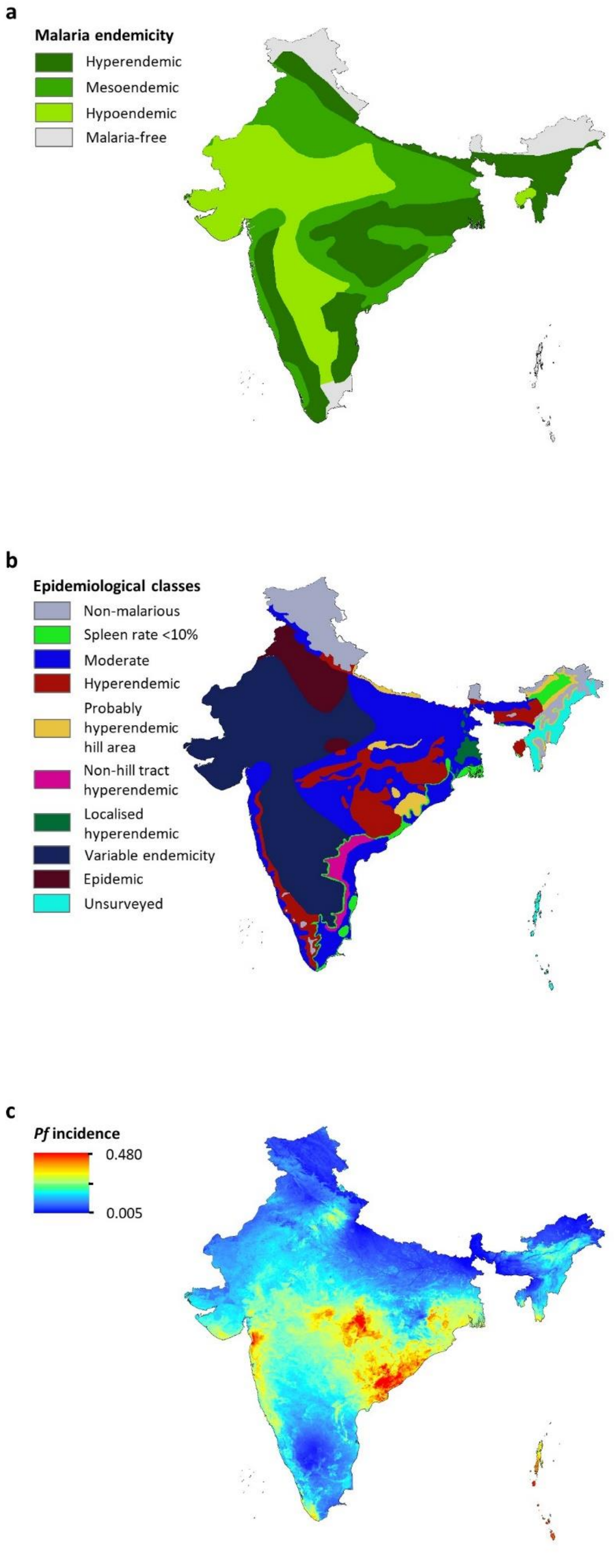
a
Malaria endemicity
Hyperendemic
Mesoendemic
Hypoendemic
Malaria-free
b
Epidemiological classes
Non-malarious
Spleen rate <10%
Moderate
Hyperendemic
Probably hyperendemic hill area
Non-hill tract hyperendemic
Localised hyperendemic
Variable endemicity
Epidemic
Unsurveyed
c
Pf incidence
0.480
0.005

Supplementary Fig. S3 (a and b) Historical and (c) contemporary maps of malaria that were used in the present analysis. (a) A map of malaria endemicity that was generated by Lysenko *et al.* (1968). Reprinted with permission from Elsevier Science Ltd. [17] (b) A map showing various epidemiological classes of malaria, as defined by Hehir (1927).[18] CC BY 4.0 (c) A model-based geostatistical map of *Plasmodium falciparum* incidence, as estimated by Gething *et al.* (2011). [19] CC BY 2.0

Other model covariates

It has been previously mentioned that β^s is particularly prevalent in STs and SCs, many of whom reside in rural areas (www.censusindia.gov.in). To see if this pattern could be captured in our model, we sourced data on two metrics for urban accessibility: (i) nighttime lights (NTL), and (ii) travel time to the nearest city of at least 50,000 people by land- or water-based travel. Data were obtained from the Defense Meteorological Satellite Program's Operational Linescan System (DMSP-OLS) (https://explorer.earthengine.google.com/) and the European Commission's Joint Research Centre Science Hub (http://forobs.jrc.ec.europa.eu/products/gam/), respectively. Both were accessed on 29 January 2018. The NTL series has a resolution of 1km x 1km and is based on the satellite detection of visible and near-infrared (VNIR) emission sources at night. The accessibility map has a resolution of 5km x 5km and incorporates information on transport networks (rail, road and sea) as well as the environmental and political factors that affect travel times between locations.

The final covariate explored in our analysis includes geographical location, given by latitude and longitude in decimal degrees (see Supplementary Information S1).

Model selection

The GAM selection process requires consideration of two key model features. First, the covariates to include in the final model must be selected, and a decision about which covariates to include as smoothing terms made. Second, given a model structure, the degree of smoothness

for each smoothing term must be optimally balanced such that under- or over-smoothing is avoided.

Prior to the model selection process, the data were split into two subsets: (i) those surveys that were carried out in scheduled populations, and (ii) those that were carried out in non-scheduled populations. Model selection was then carried for each of the two datasets separately. A backward stepwise approach was used to select the final model for each dataset. The starting model included smooth terms for latitude and longitude (both separately and in combination), historical malaria (Lysenko *et al.*, 1968), contemporary malaria, NTL and accessibility. Cubic regression splines were used for all smooth terms except the two-dimensional geographical term, for which we used thin-plate regression splines. Each survey's contribution to the model was weighted according to its survey size.

The variables associated with the highest *p*-values were successively removed until the generalized cross-validation criterion (GCV, a computationally efficient generalisation of standard out-of-sample predictive performance), Akaike Information Criterion (AIC) and mean square error (MSE) of the model were minimised.[11] The deviance explained and R^2 were also examined. Finally, smoothing parameters, which determine the degree of smoothness of the predictive functions, were estimated from the data via restricted maximum likelihood (REML).

Interpolation to generate two continuous β^S allele frequency surfaces

The final model for each data subset was run repeatedly over 2500 bootstrap samples of the dataset, generating new model parameter estimates for each bootstrapped sample. Each iteration of the model was then used to make predictions regarding β^S allele frequency at unsampled locations at a resolution of 10km x 10km. This resulted in a probability distribution of predicted

β^S allele frequency for each 10km x 10km pixel. The final β^S allele frequency maps are displayed using the central tendency (median) of these predictions.

The final β^S allele frequency maps for scheduled and non-scheduled populations represent the β^S frequency distribution when the proportion of scheduled groups in the population is 100% and 0%, respectively. This is of course an unrealistic portrayal of the situation in India; rather, a better estimate would fall somewhere between the two estimates, with the weight of each estimate being determined by the proportion of scheduled groups in the population. We obtained data on the district-wise distribution of scheduled groups from the India Census website (http://www.censusindia.gov.in/2011census/SC-ST/pca_state_distt_sc.xls and http://www.censusindia.gov.in/2011census/SC-ST/pca_state_distt_st.xls) (Figure S4) and used this to calculate weighted averages of the two estimates in each 10km x 10km pixel, using the formula:

$$E(Y)^{adj} = (E(Y)^S * p^S) + (E(Y)^{NS} * (1 - p^S))$$

where Y denotes β^S allele frequency, $E(Y)^{adj}$ denotes the adjusted predicted value of Y, $E(Y)^S$ denotes the predicted value of Y among scheduled populations, p^s denotes the proportion of scheduled groups in the population, and $E(Y)^{NS}$ denotes the predicted value of Y among non-scheduled populations. The final interpolated map of β^S in India thus represents the median of 2500 predictions, adjusted for the proportion of scheduled groups in the population.

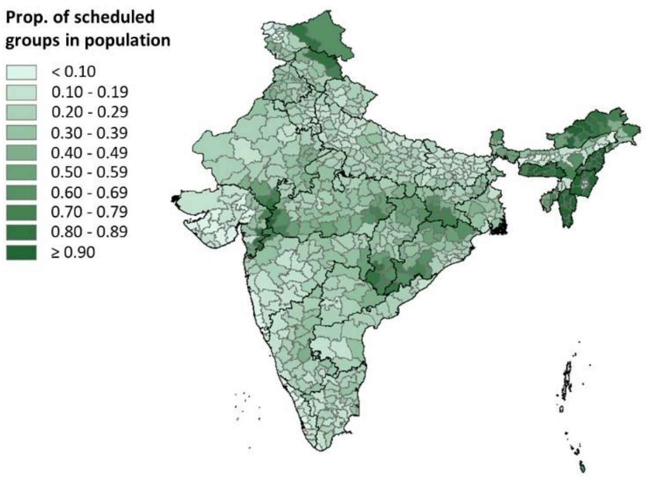

Supplementary Fig. S4 Map of the proportion of scheduled groups in the general population in each district (*n* = 666), based on data from the latest census in 2011 (www.censusindia.gov.in)

Variability in model predictions

The predictive output of the GAM of course varies between the bootstrapped samples For spatial predictions, this variability varies geographically and is a function of the quality, quantity and sample size of the available data. In addition, heterogeneity in the observed frequencies of β^S can affect the level of variability in the model's predictions. To quantify the variability in the behaviour of the GAM predicting β^S allele frequency, we calculated the 95% CI of the probability distribution of predicted β^S allele frequency for each 10km x 10km pixel.

All analyses were performed using R 3.3.2. Full scripts of the code are available on request.

The final maps

Predicted maps for scheduled and non-scheduled populations were generated separately and are shown in Supplementary Figures S5 and S6. These were paired, together with district-level data on the proportion of scheduled and non-scheduled groups in the population, to generate a composite β^S allele frequency map. This is shown in Figure 2b of the main text and displays

the median value of the 2500 bootstrapped predictions generated for each 10km x 10km pixel. The 95% CI of the bootstrapped predictions for each pixel provides a measure of how consistently the GAM performs and is shown in Supplementary Figure S7. Variability in our predictions is greatest where data are present but sparse (e.g. in northeastern Assam) and/or where there is considerable heterogeneity in the observed prevalence (e.g. along the border of Karnataka and Tamil Nadu). In these areas, variability in the model's predictions was at least 50%. High variability of up to 12% was also observed in Jammu & Kashmir, Punjab, northeastern Madhya Pradesh, West Bengal and the eastern part of Odisha, extending down into the northern districts of Andhra Pradesh and Telangana. There was low variability (<5%) across most of the central belt of high β^S frequency. However, variability was also low wherever data were completely absent. This is due to the absence of data preventing higher β^S allele frequencies from being predicted.

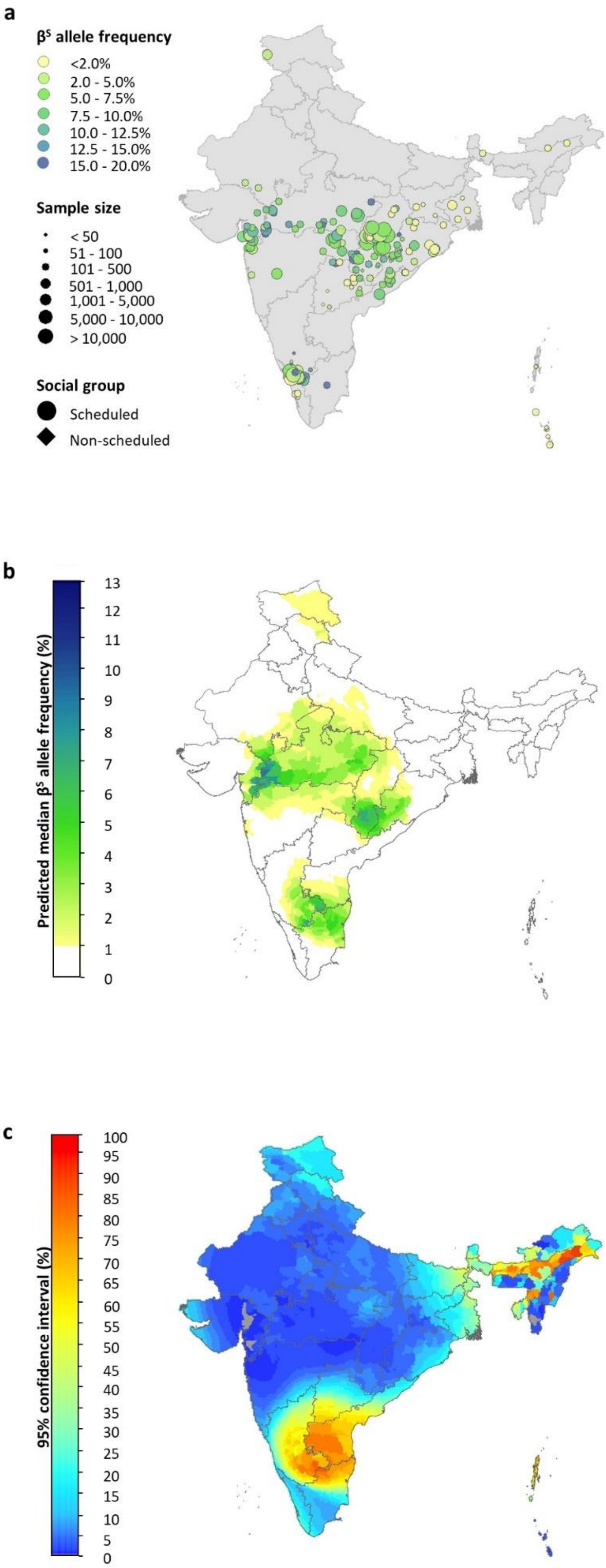
a
βS allele frequency
<2.0%
2.0 - 5.0%
5.0 - 7.5%
7.5 - 10.0%
10.0 - 12.5%
12.5 - 15.0%
15.0 - 20.0%
Sample size
< 50
51 - 100
101 - 500
501 - 1,000
1,001 - 5,000
5,000 - 10,000
> 10,000
Social group
Scheduled
Non-scheduled
b
Predicted median βS allele frequency (%)
13
12
11
10
9
8
7
6
5
4
3
2
1
0
c
95% confidence interval (%)
100
95
90
85
80
75
70
65
60
55
50
45
40
35
30
25
20
15
10
5
0

Supplementary Fig. S5 (a) A map of the sickle-cell surveys included in the scheduled population subset of our database (n = 193). Data points are coloured according to the β^S allele frequency reported in the study sample. The size of the data points corresponds to their sample size. A spatial jitter of up to 0.3° latitude and longitude decimal degrees coordinates was applied to improve visualisation of the data. (b) Map of median and (c) map of uncertainty in the predicted β^S allele frequency estimates in India at a resolution of 10km x 10km. State boundaries are displayed in grey

a
b
c

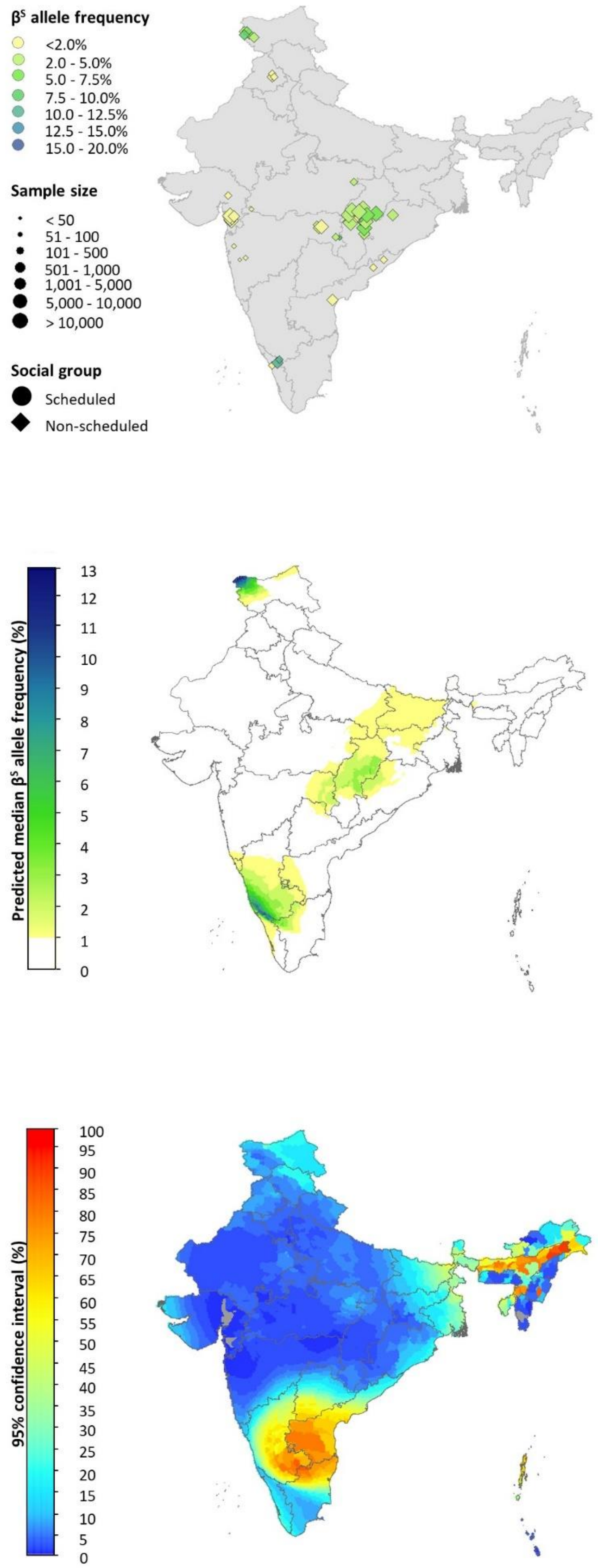
βS allele frequency
<2.0%
2.0 - 5.0%
5.0 - 7.5%
7.5 - 10.0%
10.0 - 12.5%
12.5 - 15.0%
15.0 - 20.0%
Sample size
< 50
51 - 100
101 - 500
501 - 1,000
1,001 - 5,000
5,000 - 10,000
> 10,000
Social group
Scheduled
Non-scheduled
Predicted median βS allele frequency (%)
13
12
11
10
9
8
7
6
5
4
3
2
1
0
95% confidence interval (%)
100
95
90
85
80
75
70
65
60
55
50
45
40
35
30
25
20
15
10
5
0

Supplementary Fig. S6 (a) A map of the sickle-cell surveys included in the non-scheduled population subset of our database (n = 56). Data points are coloured according to the β^S allele frequency reported in the study sample. The size of the data points corresponds to their sample size. A spatial jitter of up to 0.3° latitude and longitude decimal degrees coordinates was applied to improve visualisation of the data. (b) Map of median and (c) map of uncertainty in the predicted β^S allele frequency estimates in India at a resolution of 10km x 10km. State boundaries are displayed in grey

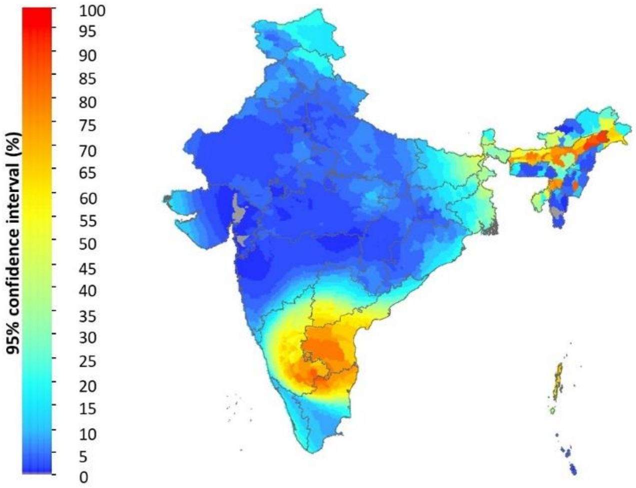

Supplementary Fig. S7 A map of the variability in the behaviour of the model predicting β^S allele frequency at each 10km x 10km pixel, given as the 95% confidence interval of 2500 bootstrapped predictions. State boundaries are displayed in grey

a

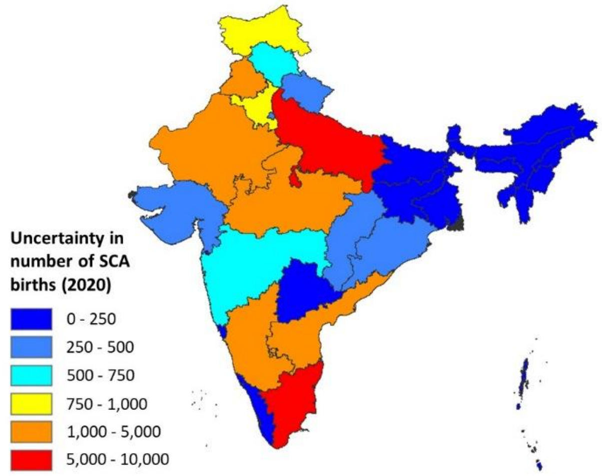

b

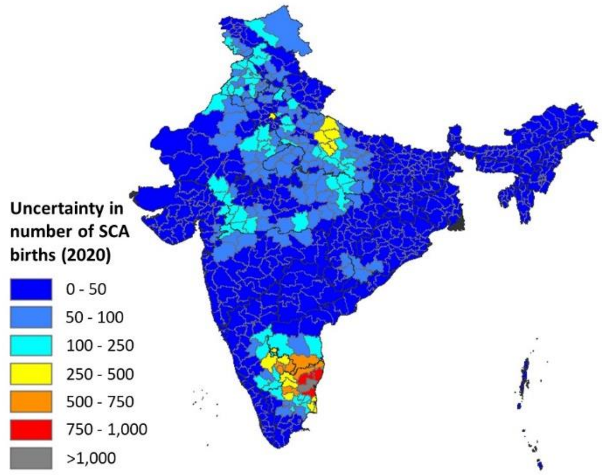

Supplementary Fig. S8 Map of the variability in estimated number of scheduled newborns born with SCA in India, by (a) state and, (b) district, in 2020. The variability is displayed as the size of the 95% confidence interval, i.e. the difference between the 97.5th and 2.5th percentiles. The states and districts shaded grey are those where our estimates were highly variable (>10 000 and >1000), respectively

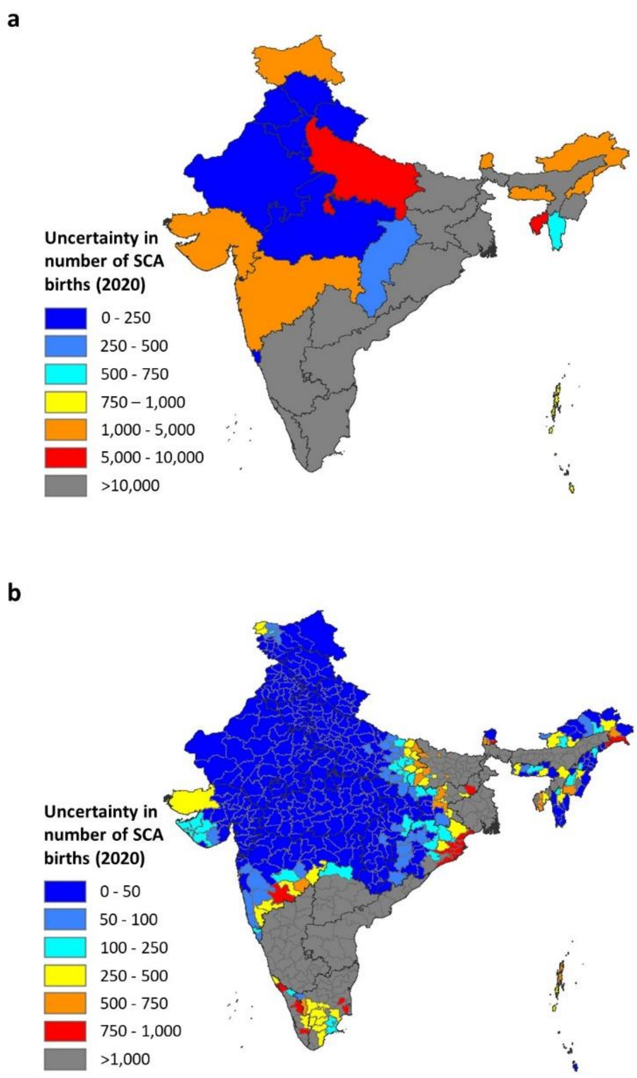

Supplementary Fig. S9 Map of the variability in estimated number of non-scheduled newborns born with SCA in India, by (a) state and, (b) district, in 2020. The variability is displayed as the size of the 95% confidence interval, i.e. the difference between the 97.5th and 2.5th percentiles. The states and districts shaded grey are those where our estimates were highly variable (>10 000 and >1000), respectively

Supplementary Information 3: Model validation

Model validation was performed for the scheduled and non-scheduled GAMs separately. To assess the model's predictive ability, a validation run was performed for each population sub-group to quantify the disparity between the model's predictions and a hold-out subset of the data. The hold-out dataset was obtained by taking a semi-random sample of the data in which there was a minimum distance of 100m between points, thereby improving the coverage of the held-out data points. The models were run with the remaining 90% of the data and their predictions compared with the observed allele frequencies from the hold-out dataset. The prediction's mean error (ME) and mean absolute error (MAE) were used to measure the model's overall bias and accuracy, respectively. The ME is the average distance between the actual data points and the predicted values. The MAE is a measure of the average magnitude of the errors in the predicted values.[1]

Supplementary Information 4: Generating newborn estimates

Whilst an allele frequency map indicates areas of high and low prevalence, it does not relay information regarding the health burden of the disorder in terms of the absolute number of newborns affected. Rather, this is dependent on a combination of allele frequency, population density and birth rate. We combined our adjusted allele frequency map with high-resolution birth count data to generate estimates for the number of newborns born with SCA in India in 2020. We used the 1km x 1km population count map from the NASA Socioeconomic Data and Applications Center (SEDAC) at www.sedac.ciesin.columbia.edu (accessed on 29 January 2018)[20] and multiplied the raster by the average crude birth rate for the 5-year periods, 2015-2020 and 2020-2025, taken from the UN world population prospects 2017 database,[21] to calculate the number of newborns in each pixel. We then rescaled the birth count data to ensure matching spatial resolution (10km x 10km) with our prediction map.

Genotype proportions were calculated from the predicted β^S allele frequency map using Hardy-Weinberg proportions. The proportion of SCA newborns were calculated as q^2, where q is the β^S allele frequency. The genotype proportions were then multiplied by birth count data for each pixel to generate estimates for the number of SCA babies born in 2020. Uncertainty in our newborn estimates stems both from uncertainty in our β^S frequency predictions and uncertainty in birth rate.

Supplementary Bibliography S1

1 Piel, F. B. *et al.* Global epidemiology of sickle haemoglobin in neonates: a contemporary geostatistical model-based map and population estimates. *Lancet* **381**, 142-151, doi:10.1016/s0140-6736(12)61229-x (2013).

2 Reich, D., Thangaraj, K., Patterson, N., Price, A. L. & Singh, L. Reconstructing Indian Population History. *Nature* **461**, 489-494, doi:10.1038/nature08365 (2009).

3 Shrikhande, A. V. *et al.* Prevalence of the beta(S) gene among scheduled castes, scheduled tribes and other backward class groups in Central India. *Hemoglobin* **38**, 230-235, doi:10.3109/03630269.2014.931287 (2014).

4 Colah, R., Mukherjee, M. & Ghosh, K. Sickle cell disease in India. *Current opinion in hematology* **21**, 215-223, doi:10.1097/moh.0000000000000029 (2014).

5 Ashley-Koch, A., Yang, Q. & Olney, R. S. Sickle Hemoglobin (Hb S) Allele and Sickle Cell Disease: A HuGE Review. *American Journal of Epidemiology* **151**, 839-845, doi:10.1093/oxfordjournals.aje.a010288 (2000).

6 Colah, R. B., Mukherjee, M. B., Martin, S. & Ghosh, K. Sickle cell disease in tribal populations in India. *The Indian Journal of Medical Research* **141**, 509-515, doi:10.4103/0971-5916.159492 (2015).

7 Naik, R. P. & Haywood, C. Sickle cell trait diagnosis: clinical and social implications. *Hematology / the Education Program of the American Society of Hematology. American Society of Hematology. Education Program* **2015**, 160-167, doi:10.1182/asheducation-2015.1.160 (2015).

8 Tubman, V. N. & Field, J. J. Sickle solubility test to screen for sickle cell trait: what's the harm? **2015**, 433-435, doi:10.1182/asheducation-2015.1.433 (2015).

9 Hardy, G. H. MENDELIAN PROPORTIONS IN A MIXED POPULATION. *Science (New York, N.Y.)* **28**, 49-50, doi:10.1126/science.28.706.49 (1908).

10 Weinberg, W. in *Jahresh Wuertt Verh Vaterl Naturkd* Ch. 64, 369-382 (1908).

11 Wood, S. *Generalized Additive Models: An Introduction with R.* (Chapman and Hall/CRC 2006).

12 Geman, S., Bienenstock, E. & Doursat, R. Neural Networks and the Bias/Variance Dilemma. *Neural Computation* **4**, 1-58, doi:10.1162/neco.1992.4.1.1 (1992).

13 Friedman, J. H. Greedy function approximation: A gradient boosting machine. *Ann. Statist.* **29**, 1189-1232, doi:10.1214/aos/1013203451 (2001).

14 Hastie, T. & Tibshirani, R. Generalized Additive Models: Some Applications. *Journal of the American Statistical Association* **82**, 371-386, doi:10.1080/01621459.1987.10478440 (1987).

15 West, R. M. in *Modern Methods for Epidemiology* (eds Yu-Kang Tu & Darren C. Greenwood) 261-278 (Springer Netherlands, 2012).

16 Piel, F. B. *et al.* Global distribution of the sickle cell gene and geographical confirmation of the malaria hypothesis. *Nature communications* **1**, 104-104, doi:10.1038/ncomms1104 (2010).

17 Lysenko, A. J. & Semashko, I. N. Geography of malaria. A medico-geographic profile of an ancient disease [in Russian]. *Itogi Nauki: Medicinskaja Geografia*, 25-146 (1968).

18 Hehir, P. *Malaria in India*, <https://wellcomecollection.org/works/axv46sh5> (1927).

19 Gething, P. W. *et al.* A new world malaria map: Plasmodium falciparum endemicity in 2010. *Malaria Journal* **10**, 378-378, doi:10.1186/1475-2875-10-378 (2011).

20 Center for International Earth Science Information Network - CIESIN - Columbia University. (NASA Socioeconomic Data and Applications Center (SEDAC), Palisades, NY, 2017).

21 Affairs, U. N. D. o. E. a. S. World population prospects, the 2017 Revision. (United National Population Division, New York, 2017).

Supplementary Bibliography S2: Sickle-cell prevalence surveys

The sources include in the final database are:

1 Balgir, R. S. Clinical and hematological profiles of hemoglobinopathies in two tribal communities in Sundargarh district in Orissa, India *Int. J. Hum. Gen.* **3** (2003).

2 Balgir, R. S. A cross-sectional study of hemoglobin disorders in pregnant women attending two Urban hospitals in eastern coast of Odisha, India. *Online J. Health Allied Sci.* **12** (2013).

3 Bhukhanvala, D. S. *et al.* Hemoglobin variants in Muslim community in South Gujarat, Western India. *International journal of laboratory hematology* **36**, e15-e17, doi:10.1111/ijlh.12123 (2014).

4 Bhukhanvala, D. S. *et al.* Antenatal screening for identification of couples for prenatal diagnosis of severe hemoglobinopathies in Surat, South Gujarat. *J. Obstet. Gynecol. India* **63**, 123-127, doi:10.1007/s13224-012-0271-4 (2013).

5 Bhukhanvala, D. S., Sorathiya, S. M., Shah, A. P., Patel, A. G. & Gupte, S. C. Prevalence and hematological profile of beta-thalassemia and sickle cell anemia in four communities of Surat city. *Indian journal of human genetics* **18**, 167-171, doi:10.4103/0971-6866.100752 (2012).

6 Centre, S. R. K. R. Prevalence of β-thalassaemia trait (BTT) and sickle cell trait (SCT) in the Surat population. (Surat Raktadan Kendra & Research Centre, 2008).

7 Centre, S. R. K. R. β-thalassaemia and sickle cell trait in Muslim, Patel and Ghanchi communities of Surat. (Surat Raktadan Kendra & Research Centre, 2008).

8 Chakrabarti, S., Mandal, K., Pathak, S., Patra, A. & Pal, S. Haemoglobinopathies among the tribal and non-tribal antenatal mothers in a tertiary care hospital of rural West Bengal, India. *Bangladesh J. Med. Sci.* **15**, 90-94 (2016).

9 Chirmulay, D., Kate, S. L., Mokashi, G. D. & Bankar, M. P. Prevalence of sickle cell haemoglobin in Vansda district Valsad Gujarat: A pilot study *Indian Journal of Hematology & Blood Transfusion* **12** (1994).

10 Choubisa, S. L. Abnormal Haemoglobins, Thalassaemia and G-6-Pd Enzyme Deficiency in Rajasthan (Western-India) *Haematologica* **24**, 153-165 (1991).

11 Choubisa, S. L. Sickle cell haemoglobin, thalassaemia and G-6-PD enzyme deficiency genes in Garasiya tribe inhabited malaria endemic areas of Sirohi District, Rajasthan (India). *J Commun Dis* **41**, 13-18 (2009).

12 Choudhuri, S., Sen, A., Ghosh, M. K., Misra, S. & Bhattacharyya, M. Effectiveness of Prenatal Screening for Hemoglobinopathies in a Developing Country. *Hemoglobin* **39**, 380-383, doi:10.3109/03630269.2014.1003564 (2015).

13 Deore, A. U. & Urade, B. P. Incidence of sickle cell trait among the Mahar of Raipur, Chhattisgarh. *Anthropologist* **15**, 377-378 (2013).

14 Dey, A. in *National Seminar on Mother Child Health Status & XII Annual Conference of Indian Society of Human Genetics* (Calcutta, 1987).

15 Dixit, S., Sahu, P., Kar, S. K. & Negi, S. Identification of the hot-spot areas for sickle cell disease using cord blood screening at a district hospital: an Indian perspective. *Journal of community genetics* **6**, 383-387, doi:10.1007/s12687-015-0223-7 (2015).

16 Dolai, T. K., Dutta, S., Bhattacharyya, M. & Ghosh, M. K. Prevalence of hemoglobinopathies in rural Bengal, India. *Hemoglobin* **36**, 57-63, doi:10.3109/03630269.2011.621007 (2012).

17 Fareed, M., Anwar, M. A., Ahmad, M. K. & Afzal, M. Gene frequency reports of sickle cell trait among six human populations of Jammu and Kashmir, India. *Gene Rep.* **4**, 1-5, doi:10.1016/j.genrep.2016.02.003 (2016).

18 Feroze, M. in *National Conference on Hemoglobinopathies* (Bangalore, 2013).
19 Fulmali, P. M., Kate, S. L., Bankar, M. P., Mokashi, G. D. & Phadke, M. A. in *National Seminar on Mother Child Health Status & XII Annual Conference of Indian Society of Human Genetics* (Calcutta, 1987).
20 Godbole, S. *et al.* in *Proceedings of National Symposum on Tribal Health* (eds N. Singh *et al.*) (Jabalpur, 2006).
21 Gunjal Sandeep, S., Narlawar Uday, W., Humne Arun, Y. & Chaudhari Vijaya, V. L. Prevalence of sickle cell disorder and anaemia in tribal school students from central India. *Int. J. Collab. Res. Intern. Med. Public Health* **4**, 1321-1329 (2012).
22 Italia, Y. *et al.* Feasibility of a Newborn Screening and Follow-up Programme for Sickle Cell Disease among South Gujarat (India) Tribal Populations. *Journal of medical screening* **22**, 1-7, doi:10.1177/0969141314557372 (2015).
23 Jain, M. K., Padmanabham, P. B. S. V., Dhar, P., Das, K. & Rao, V. R. in *International seminar on Haemoglobinopathies South Asia Problems and Prevention* (Calcutta, 2000).
24 Kaur, M., Dangi, C. B. S. & Singh, H. To study the haemoglobinopathies and ratio of copper and zinc in Sindhi Community of Bhopal. *Int. J. Pharma Bio Sci.* **4**, 672-691 (2013).
25 Kaur, R. & Kaur, H. Haemoglobin Variants In Some Ethnic Groups Of Punjab (North India). *Indian journal of human genetics* **5**, 57-60 (1999).
26 Khurana, P., Aggarwal, A., Huidrom, S. S. & Kshatriya, G. K. Haptoglobin polymorphism among the tribal groups of southern Gujarat. *Indian journal of human genetics* **17**, 169-174, doi:10.4103/0971-6866.92096 (2011).
27 Mission, N. R. H. Sickle Cell Disease Control Program. (Maharashtra, 2016).
28 Mohanty, D. Current status of sickle cell disease in India: how can you attenuate? *Molecular cytogenetics* **7**, I45, doi:10.1186/1755-8166-7-s1-i45 (2014).
29 Mohanty, D., Mohanty, N. & Kate, S. L. Sickle Cell Anaemia, Thalassemia and Other Genetic Problems Amongst Tribals of Maharashtra (Indian Council of Medical Research, 1995).
30 Mukhopadhyay, D. *et al.* Spectrum of Hemoglobinopathies in West Bengal, India: A CE-HPLC Study on 10407 Subjects. *Indian journal of hematology & blood transfusion : an official journal of Indian Society of Hematology and Blood Transfusion* **31**, 98-103, doi:10.1007/s12288-014-0373-5 (2015).
31 Nagar, R., Sinha, S. & Raman, R. Haemoglobinopathies in eastern Indian states: a demographic evaluation. *Journal of community genetics* **6**, doi:10.1007/s12687-014-0195-z (2015).
32 Nimgaonkar, V., Krishnamurti, L., Prabhakar, H. & Menon, N. Comprehensive integrated care for patients with sickle cell disease in a remote aboriginal tribal population in Southern India. *Pediatr. Blood Cancer* **61**, 702-705, doi:10.1002/pbc.24723 (2014).
33 Panigrahi, S., Patra, P. K. & Khodiar, P. K. Neonatal screening of sickle cell anemia: A preliminary report. *Indian journal of pediatrics* **79**, 747-750, doi:10.1007/s12098-011-0682-8 (2012).
34 Panigrahi, S., Patra, P. K. & Khodiar, P. K. The screening and morbidity pattern of sickle cell anemia in chhattisgarh. *Indian journal of hematology & blood transfusion : an official journal of Indian Society of Hematology and Blood Transfusion* **31**, 104-109, doi:10.1007/s12288-014-0407-z (2015).
35 Patel, A. G., Shah, A. P., Sorathiya, S. M. & Gupte, S. C. Hemoglobinopathies in South Gujarat population and incidence of anemia in them. *Indian journal of human genetics* **18**, 294-298, doi:10.4103/0971-6866.107979 (2012).

36 Patel, J., Patel, B., Gamit, N. & Serjeant, G. R. Screening for the sickle cell gene in Gujarat, India: A village-based model. *Journal of community genetics* **4**, 43-47, doi:10.1007/s12687-012-0116-y (2013).
37 Patra, P. K. & al., e. *Sickle Cell Screening Project - At A Glance*, <www.scic.cg.nic.in> (2016).
38 Patra, P. K., Khodiar, P. K., Hambleton, I. R. & Serjeant, G. R. The Chhattisgarh state screening programme for the sickle cell gene: a cost-effective approach to a public health problem. *Journal of community genetics* **6**, 361-368, doi:10.1007/s12687-015-0222-8 (2015).
39 Purohit, P., Dehury, S., Patel, S. & Patel, D. K. Prevalence of deletional alpha thalassemia and sickle gene in a tribal dominated malaria endemic area of eastern India. *ISRN hematology* **2014**, 745245, doi:10.1155/2014/745245 (2014).
40 Qamra, S., Roy, J. & Srivastava, P. Impact of sickle cell trait on physical growth in tribal children of Mandla district in Madhya Pradesh, India. *Annals of human biology* **38**, 685-690, doi:10.3109/03014460.2011.608378 (2011).
41 Research, I. C. o. M. Jai Vigyan S & T Mission Project on Community Control of Thalassaemia Syndromes – Awareness, Screening, Genetic Counselling and Prevention: A National Multicentric Task Force Study of ICMR (Indian Council of Medical Research, India, 2008).
42 Research, I. C. o. M. Intervention Programme for Nutritional Anaemia and Haemoglobinopathies against some Primitive Tribal Populations of India: A National Multicentric Study of ICMR. (Indian Council of Medical Research, India, 2010).
43 Rupani, M. P., Vasava, B. C., Mallick, K. H., Gharat, V. V. & Bansal, R. Reaching community through school going children for sickle cell disease in Zankhvav Village of Surat District, Western India. *Online J. Health Allied Sci.* **11** (2012).
44 Sachi Devi, S. & Naidu, J. M. in *Proceedings of XII Annual Conference of Indian Society of Human Genetics* (eds S. B. Roy, A. Chowdhury, & B. Sarkar) (1987).
45 Sahoo, S. S., Biswal, S. & Dixit, M. Distinctive mutation spectrum of the HBB gene in an urban eastern Indian population. *Hemoglobin* **38**, 33-38, doi:10.3109/03630269.2013.837394 (2014).
46 Sathe, M. *et al.* in *Proceedings of XII Annual Conference of Indian Society of Human Genetics* (eds S. B. Roy, A. Chowdhury, & B. Sarkar) (1987).
47 Shah, S. P. *et al.* Effectiveness and Feasibility of Weekly Iron and Folic Acid Supplementation to Adolescent Girls and Boys through Peer Educators at Community Level in the Tribal Area of Gujarat. *Indian journal of community medicine : official publication of Indian Association of Preventive & Social Medicine* **41**, 158-161, doi:10.4103/0970-0218.173498 (2016).
48 Shrikhande, A. V. *et al.* Prevalence of the βs gene among scheduled castes, scheduled tribes and other backward class groups in central India. *Hemoglobin* **38**, 230-235 (2014).
49 Singh, M. P. S. S. *et al.* in *Proceedings of National Symposum on Tribal Health* (eds N. Singh *et al.*) (Jabalpur, 2006).
50 Urade, B. P. Haemoglobin S and βThal: Their distribution in Maharashtra, India. *Int. J. Biomed. Sci.* **9**, 75-81 (2013).
